# “Kickstart to Recovery”: An Irish Football Program for Mental Health Service Users

**DOI:** 10.1177/15394492231177281

**Published:** 2023-06-23

**Authors:** Hannah Casey, Jenny Johnston, Maurice Dillon, Clodagh Nolan

**Affiliations:** 1 Trinity College Dublin, Ireland; 2 Dublin South Central Mental Health Service, Crumlin, Ireland; 3 Louth Meath Mental Health Service, Ireland

**Keywords:** mental health, exercise, evidence-based practice, quality of life

## Abstract

The “Kickstart to Recovery” program is a collaboration between Irish mental health occupational therapists and the Football Association of Ireland. This pilot study aimed to investigate whether participants experienced changes in quality of life, recovery, social gains, and the meaning of football following participation in the program. A quantitative pre–post study design was employed, with 27 participants completing a questionnaire consisting of outcome measures aimed to measure the above changes. Findings revealed statistically significant improvements in the Short Form 36 Health Survey Questionnaire (SF-36) “Energy/Fatigue” domain for the total sample and the Recovery Assessment Survey–Domains and Scales (RAS-DS) “Mastering My Illness” domain for first-time program participants. Statistically significant results were found for domains of “Social Functioning” and “Emotional Wellbeing” in groups incorporating additional social elements. The Engagement in Meaningful Activities Survey (EMAS) showed no change for the personal meaning participants attributed to football; however, high pre-test scores were noted. The “Kickstart to Recovery” program is attributed as a possible factor contributing to these results.

## Introduction

In recent years, the interconnection between mental and physical health has become more prominent. There is growing recognition of the benefits of physical activity on mental health and the negative impact mental health challenges can have on physical health.

In an Irish context, research by Matthews et al. (2018) found that 72% of individuals with mental health challenges did not meet recommended physical activity guidelines. In addition to common barriers such as lack of time and motivation, low mood, stress, and lack of social support are found to significantly hinder opportunities to meet these guidelines (Firth et al., 2017).

Physical activity counteracts negative health problems, with benefits such as improved cognitive functioning (Firth et al., 2017), management of symptoms associated with psychotic disorders (Firth et al., 2015), mood, concentration, and sleep patterns (Alexandratos et al., 2012). Team sports that include interactions with others can provide recovery-oriented benefits, such as social interaction and building a sense of self (Carless & Douglas, 2008).

### Physical Activity and Occupational Therapist’s in Mental Health Services

In Ireland, there is currently no mental health professional within the multidisciplinary team designated to the promotion and delivery of physical activity interventions for clients (Matthews et al., 2018). As occupational therapy is grounded in the “belief that active engagement in occupation promotes, facilitates, supports, and maintains health and participation” (American Occupational Therapy Association [AOTA], 2014, p. S4), occupational therapists (OTs) are suitably positioned to bridge the gap and promote engagement and participation in physical activity to support well-being. OTs have an understanding of both the health benefits highlighted above and the importance of “engaging in,” not only “participating in” an activity, which has the potential to influence how physical activity interventions are developed and delivered in mental health services (Cole, 2014).

### Benefits of Football for Mental Health Service Users

Physical activity that promotes a recovery-orientated approach has the greatest value to mental health services. Football provides both social and physical elements, and hence has become increasingly popular as an intervention method in mental health services (Benkwitza & Healy, 2019).

Physical health and fitness improvements act as motivational factors for partaking in the football programs (Friedrich & Mason, 2018; Hargreaves & Pringle, 2019; McArdle et al., 2012). In Moloney and Rohde’s (2017) initial evaluation of “Kickstart to Recovery” (formally “Kickstarting Recovery”), participants reported to have more energy, improved fitness, and better sleep as a result of participation in the weekly session. Improvements in performance capacity of skills and abilities of the players were also noted (Moloney & Rohde, 2017).

In addition to physical health benefits, participation in football programs can support mental health recovery. An element of recovery is developing skills and strategies to manage mental health symptoms. Research on football programs for mental health service users reported development of coping skills which act as a strategy in itself (Darongkamas et al., 2011; Friedrich & Mason, 2018). Hargreaves and Pringle (2019) found that participants used self-talk, peer support, and goal setting as strategies for self-control management. Both Moloney and Rohde (2017) and Lamont and colleagues (2017) identified participants’ use of football as a distraction technique for unhelpful thoughts.

Social gains identified in previous studies are noted as a significant outcome of participation in football programs as part of mental health services. These social gains included connecting with others, improving social confidence (Butterly et al., 2006), building a social identity, reducing social isolation (Benkwitza & Healy, 2019), and increased feeling of belonging and acceptance (Darongkamas et al., 2011). Benkwitza and Healy (2019) found the football intervention to be a medium for service users to improve their active citizenship role as it allowed for exploration of their community and sourcing ways to contribute to them. Ramon (2018) describes active citizenship as a strong element in improving social recovery and hence benefits both the person and the community.

On the contrary, Butterly and colleagues (2006) found a lack of an “exercise buddy” can act as a barrier to being active. Hargreaves and Pringle (2019) found that social support acts as a means of overcoming barriers to engagement and reduces participant’s reliance on mental health service staff to accompany them to facilities. This again highlights the benefit of the team sport element of football.

In addition to these social benefits, the programs also created a safe social environment for participants. Trauma-informed care is an approach, based on the knowledge of understanding the impact of trauma on service users and should be applied when working with all mental health service users to prevent the risk of re-traumatization (Harris & Fallot, 2001). Safety and peer support are two core principles of trauma-informed care (Champagne, 2020). Both Darongkamas and colleagues (2011) and Moloney and Rohde (2017) identified the intervention environments as safe places, where service users felt the football created a normal environment and were comfortable enough to encourage others to join. Within the safe environment, peer support developed beyond that on the football field. McArdle and colleagues (2012) found participants perceived everyone in the group to be present for similar reasons, which opened discussions beyond football to that of personal mental health issues and mutual understanding.

### Football and Mental Health Services in an Irish Context: The “Kickstart to Recovery” Program

Both the Football Association of Ireland (FAI) and Health Service Executive (HSE) highlighted the need for mental health support initiatives to be developed. HSE mental health services required community-based practice that supported recovery and physical activity. Similarly, the FAI sought to expand their “Football for All” initiative to further populations experiencing disability. To meet both needs, HSE occupational therapy and the FAI National Development Coordinator collaborated. “Kickstart to Recovery” was formed with the aim of promoting improvements in social aspects such as community integration, reduced isolation and social skills, prompting the benefits of physical activity, mental health recovery, quality of life, and creating a fun and supportive environment. The program was piloted in 2011, running for 4 weeks in a single site. The success of the pilot led to the expansion of the program to all North Dublin City Mental Health Services in 2013. As of 2017, there were 15 programs running throughout the Republic of Ireland (FAI, 2017).

Guidelines for the structure of the program are outlined in an internal “handbook” designed in 2017 by an Irish community mental health service who act as stakeholders for the program on behalf of the HSE. According to the handbook, there are 6 to 8 weeks of sessions per block, which are collaboratively planned and facilitated by the FAI coach and HSE OT. The sessions may vary with coaches’ style of training and service users’ needs. They commonly include a warm up, skill building drills, teambuilding drills, a short match, and a cool down. The active involvement of the OT in the sessions is stated in the handbook, aiming to promote co-occupation experiences for service users through opportunities for social engagement (Pierce, 2003). The OT often will have a goal setting session with each participant prior to commencing the program and support them to meet this goal throughout the program. Working with the football coach, the OT would support adaptions and grading the sessions as appropriate (Moloney & Rohde, 2017). Examples of this include supporting the coach to adapt communication methods, grading tasks to just the right challenge level, and including more social opportunities within the session. The program does not have restrictions on service users participating in multiple blocks of the program.

### Aims of the Study

To investigate the impact of the “Kickstart to Recovery” program in an Irish context, the HSE and FAI collaborated with Trinity College Dublin, providing resources for a pilot study to be conducted on the program. To provide data that would benefit the practice of the program, the aims of this research study were derived from the aims of the program. These included investigating if service users experienced a change in aspects of quality of life, recovery, or social gains following participation in the “Kickstart to Recovery” program. In addition, this study aimed to investigate if the services users experienced a change in the meaning of football to them following the participation in the Kickstart to Recovery program.

## Method

### Study Design

This study utilized quantitative pre–posttest research method using a paper-based questionnaire. The study design included a three-part process: first, the participants completed a questionnaire immediately before commencing the “Kickstart to Recovery” program, then the football program is run as normal, and finally they complete the same questionnaire on the final day of the program.

Service users from seven community mental health services were recruited for the study. These sites spread across the Republic of Ireland, situated in both rural and urban settings. To be included in the research, the participant was required to be approved to partake in the “Kickstart to Recovery” program, attended at least one session of the program, and had to complete both the pre- and post-questionnaires. Those who did not satisfy the inclusion criteria were not included in the research. Those eligible for the study were provided with a Participant Information Leaflet and given the opportunity to ask questions to ensure informed consent was provided.

### The Questionnaire

The questionnaire contained three outcome measures and demographic questions related to diagnosis, age, and gender. It included the following outcomes measures: Short Form 36 Health Survey Questionnaire (SF-36, Ware & Sherbourne, 1992), which measured quality of life; Recovery Assessment Survey–Domains and Scales (RAS-DS, Hancock et al., 2015) which measured self-reported recovery-oriented change; and the Engagement in Meaningful Activities Survey (EMAS) adapted with permission from the author for football (Goldberg et al., 2002), which measured the meaning of football for the participant.

All outcome measures were chosen for their good psychometric properties. Both the RAS-DS and SF-36 have shown to be sensitive to change over time and have produced reliable and valid mental health recovery results (Hancock et al., 2015; Ware, 2000). The EMAS test–retest reliability was shown to be acceptable when used with individuals with persistent mental health difficulties (Goldberg et al., 2002).

The RAS-DS and SF-36 consist of different domains relevant to the umbrella subject of measurement of the assessment. For the purpose of this article, the domains that will be discussed in detail are the “Mastering My Illness” of the RAS-DS and the “Energy and Fatigue,” “Social Functioning,” and “Emotional Wellbeing” of the SF-36.

### Ethical Considerations

Ethical approval was sought and granted by Trinity College Dublin Research Ethics Committee for the study. As per local requirements, additional ethical approval was granted from three separate HSE Research Ethics Committees.

### Data Analysis

Data were analyzed using descriptive statistics, and statistical analysis was conducted using nonparametric tests. The data used violated more than one assumption of parametric methods; therefore, nonparametric methods were used in this study. The factors that allow for nonparametric methods to be used are the following: sample size of <30 (*n* = 27) (Pallant, 2016), a population with a skewed distribution, and use of ordinal measures. Statistical testing was completed using IBM SPSS, v26. Two different nonparametric tests were used: the Wilcoxon signed rank test and the Kruskal–Wallis test. Wilcoxon signed rank test is a nonparametric test used to compare change in repeated measures, appropriate for testing pre- and post-test data. The Kruskal–Wallis test is a nonparametric test used to compare changes between three or more different groups. This was used to compare groups depending on the exposure to the program (number of sessions in each program). Statistical significance was initially set at *p* < .05 for all tests. As a number of tests were being conducted, the Bonferroni correction method was applied to control for Type I error (Napierala, 2012) This is found by dividing the original *p* value (.05) by the number of comparisons being made. The adjusted *p* values are noted with the original *p* value for each set of testing in the “Results” section.

## Results

Initial number of participants was 40 across the seven sites, of which 27 completed both pre-test and post-test questionnaires. All participants were male and had played football previously, approximately half at club level (55.5%). The age ranged between 18 and 58. Of the sessions offered to them, most of the participants (77.8%) attended the majority of the sessions (75% or more sessions offered).

The aim of this study was to investigate changes in quality of life, recovery, social domains, or meaning of football. The RAS-DS and EMAS provide a total score. The SF-36 does not have a total score and consists of sub-domains only. The overall pre- and post-scores of the RAS-DS and EMAS outcome measure used in the questionnaire were compared using a Wilcoxon signed rank test. The adjusted *p* value with the Bonferroni correction is *p <* .025. No significant differences were found in the overall scores, as the scores were greater than both the original (.05) and the corrected significance levels (Table 1). This was also completed for the sub-categories of first-time program participants and return program participants. No significant difference was found in the results (Table 1).

**Table 1.** Pre-Test Versus Post-Test Total Scores of the RAS-DS and EMAS Comparing Before and After Participation in the “Kickstart to Recovery”.

Total score of outcome measure tested using the Wilcoxon signed rank test	All participants	First-time participants	Returning participants
RAS-DS	.989	.075	.513
EMAS	.862	.715	.795

*Note.* RAS-DS = Recovery Assessment Survey–Domains and Scales; EMAS = Engagement in Meaningful Activities Survey.

The original significance level is *p* < .05. The significance level with Bonferroni correction applied is *p <* .025.

On further analysis of pre-test scoring for the EMAS, high initial EMAS scores were noted. This provided a small window for improvement in the post-test stage, thus imposing a potential ceiling effect (Cummingham et al., 2013).

The RAS-DS has four sub-domains and the SF-36 has nine sub-domains. Table 2 displays the results of the Wilcoxon signed rank tests used to compare the total participants, first-time participants, and the returning participants’ pre- and post-questionnaire scores for each domain. The adjusted significance level following the Bonferroni Correction was *p* < .0038. With this significance level, no statistically significant results are found.

**Table 2.** Pre-Test Versus Post-Test Scores of Each Sub-Domain of the RAS-DS and SF-36 Comparing Before and After Participation in the “Kickstart to Recovery”.

Outcome measure	Domain of outcome measure tested using the Wilcoxon signed rank test	All participants	First-time participants	Returning participants
		*p*	*p*	*p*
RAS-DS	Mastering my illness	.346	.046*	.795
RAS-DS	Looking forward	.667	.458	.506
RAS-DS	Doing things I value	.886	.104	.532
RAS-DS	Connecting and belonging	.162	.854	.144
SF-36	Physical functioning	.249	.686	.122
SF-36	Role limitation due to physical functioning	.342	1.00	.298
SF-36	Role limitation due to emotional problems	.086	.102	.291
SF-36	Energy/Fatigue	.011*	.072	.057
SF-36	Emotional well-being	.055	.257	.107
SF-36	Social functioning	.145	.276	.328
SF-36	Pain	.268	.109	.858
SF-36	General health	.464	.176	.924
SF-36	Health changes	.136	.317	.248

*Note.* RAS-DS = Recovery Assessment Survey–Domains and Scales; SF-36 = Short Form 36 Health Survey Questionnaire.

The significance level is *p* < .05. Statistical significance indicated by *. The significance level with Bonferroni correction applied is *p* < .0038. The above indicated are not statistically significant under this level.

When *p* < .05, a Wilcoxon signed rank test revealed a statistically significant difference between the pre- and post-test domain of “Energy/Fatigue” for the total participants, both those participating in the “Kickstart to Recovery” program for the first time and those returning with *p* = .011 (Table 2).

Also when *p* < .05, a Wilcoxon signed rank test found a statistically significant difference in the pre- and post-test domain of “Mastering My Illness” for those who were participating in the “Kickstart to Recovery” program for the first time, with *p* = .042 (Table 2).

Following the Wilcoxon signed rank tests, all other domains did not have a statistically significant difference in the pre- and post-tests of any RAS-DS or SF-36 sub-domains for those participating in the “Kickstart to Recovery” program in total, for the first time, or returning to the program for both the original and adjusted *p* value (Table 2).

Each site was grouped according to program duration to measure the impact of exposure to the program. Group 1 (G1) included groups with five or six sessions, Group 2 (G2) included those with seven sessions, and Group 3 (G3) those with eight sessions. The post-test comparison of the three groups, using the Kruskal–Wallis Test, was conducted on the total EMAS and RAS-DS scores and for each domain of the SF-36 and RAS-DS. As 15 tests were performed, the adjusted Bonferroni correction significance level was *p* < .0017. No statistically significant results were found with this *p* value.

When *p* < .05, two results found statistically significant differences for G1 compared with G2 and G3: “Social Functioning” (*p* = .020) and “Emotional Wellbeing” (*p* = .016). All other Kruskal–Wallis tests conducted found no statistically significant differences between the three groups under either the original or adjusted *p* values.

The relationship between these sites compared with the other sites was investigated further using information from the on-site OT’s session records and the researcher’s reflective diary. The sites in G1 were found to have social aspects embedded into the program, while no such social aspect was identified in the other sites in G2 or G3. The author recognizes the limitations posed by the small sample size, and results are indications of possible differences between the groups.

## Discussion

This research investigated if change occurred after participation in the “Kickstart to Recovery” program in the following domains: quality of life, recovery orientation, social gains, and the meaning of football for mental health service users. No results were found to be statistically significant when the Bonferroni Correction was applied. The following discussion focuses on the results with the original *p* < .05.

### Energy Levels

No statistically significant results were found in comparing pre- and post-program scores in any domain of the SF-36, except that of the “Energy Levels and Fatigue” domain. This domain captures the subjective well-being of the individual, has good empirical validity, and is the only domain that has significant correlation with both mental health and physical health (Ware, 2000; Ware & Sherbourne, 1992). The median of the pre-test (*Mdn* = 45) was on the lower level of the scores, but the post-test median (*Mdn* = 57.5) moves toward the higher scores, which would indicate that participants experienced lower levels of energy prior to commencing the program. However, given the short timeframe (between 5 and 8 weeks), the potential for improvement was demonstrated (Ware & Sherbourne, 1992). This result includes first-time participants and those returning to the program, and hence provides an indication that the “Kickstart to Recovery” program has the potential to improve energy levels and reduce fatigue on an ongoing basis. As fatigue has been shown to be a barrier to physical activity for people with mental illness in Ireland (Matthews et al., 2018), the program has the potential to aid service users in overcoming fatigue as a barrier to participating in other physical activities. This quantitative result supports the qualitative findings of Butterly and colleague’s (2006) MUSCSEL project and the “Kickstarting Recovery” program research findings of Moloney and Rohde (2017). Both studies found participants reporting increased energy levels after participation in the football programs. This benefit was found to have a positive impact upon their everyday lives, in addition to their performance during football sessions (Butterly et al., 2006).

### Mastering Illness

The results of this study found statistically significant improvement in “Mastering My Illness” for those whose first time it was to partake in the “Kickstart to Recovery” program. It did not show significance for the overall sample nor for those who had previously participated in the program. This signifies the potential initial impact of the program on the development of mental health symptom management, but has not indicated an ongoing effect on the development of these coping strategies.

Questions posed in this domain of the RAS-DS included managing symptoms, developing coping strategies, learning to detect warning signs of becoming unwell, and developing an action plan for potential relapse. This quantitative result supports a number of qualitative findings of other football interventions. In the qualitative study of the “Kickstarting Recovery” program, participants found they could use the football as a distraction technique for mental health symptoms such as unhelpful thoughts (Moloney & Rohde, 2017). Friedrich and Mason’s (2018) participants developed new coping mechanisms, reducing their anxiety as a result of their participation in a football program. Similarly, Darongkamas and colleagues (2011) found that the football program helped their participants to manage their mental health difficulties. Furthermore, Hargreaves and Pringle’s (2019) quantitative work also identified self-control strategies developed as a result of self-talk and utilizing social support. This study adds to the body of research, supporting the evidence that football programs for mental health service users have the potential to aid the participants to take control of their mental health challenges.

### Social Aspect

This study found statistically significant differences between post-program scores in both domains of “Social Functioning” and “Emotional Wellbeing” for Group 1 compared with that of Groups 2 and 3. As highlighted above, on investigation, the presence of an additional social aspect to each program was identified as a common thread which was not present in the other groups in the study. One site had refreshments after each football session, while the second site had picnic tables on-site where the service users would gather before and/or after the session to chat. The OT would sit and engage with the service users in an informal manner at these times, promoting social interaction between all service users. As identified in Moloney and Rohde’s (2017) study, football could be the bridge to this interaction, providing a topic of conversation or opportunity to reflect on the session. This social aspect was previously incorporated into the program, with Moloney and Rohde (2017) identifying it as an important part of the social environment; however, in more recent interpretations of the program, it does not appear to have been present.

This study investigates two social elements, Connecting and Belonging of the RAS-DS and Social Functioning of the SF-36, providing further understanding of this result. “Connecting and Belonging” domain focuses on community friendships (Hancock et al., 2015) while “Social Functioning” domain reflects the degree the person’s health affects their normal social activities (Ware, 2000). As such, “Connecting and Belonging” reflects social growth, while “Social Functioning” reflects social maintenance. While growing social connections is important to integrate into the community, it would be difficult to maintain these new social connections if an individual’s physical and mental health interfered significantly with their participation. Therefore, this finding indicates that if a social aspect is embedded in the sessions, the supports offered by the “Kickstart to Recovery” program may set a foundation for social maintenance.

Repper and Perkins (2003) state that social functioning and emotional well-being are important aspects of the recovery process, are “intimately interlinked” (p. 136), and need to be fostered. This is achieved in the “Kickstart to Recovery” program by embedding the social aspect into the sessions. OTs facilitating the program play a vital role in supporting this and adapting to the needs of their service users. The sessions provided a shared experience centered on the common entity of football. These experiences may provide inspiration for conversation between service users, supporting Moloney and Rohde’s (2017) findings, which allows for relationships between individuals at different stages of recovery to occur. In addition, this would support the fostering of hope as service users gain role models and peer support from each other, similar to that found by McArdle and colleagues (2012), supporting trauma-informed care principles (Champagne, 2020). This finding supports current evidence for social aspects within football-based mental health interventions, supports the recovery process, and provides strong justification for the implementation of a social aspect into the structure of the “Kickstart to Recovery” program.

### Meaning of Football

This investigation identified that all scores related to the questions on the meaning of football in the pre-test were high (ranging from 77% to 93%). When scores are this high prior to commencing the program, they may create a ceiling effect, which allows for a smaller margin of improvement to be possible at post-program stage (Cummingham et al., 2013). This indicated that football was meaningful to participants prior to commencing or returning to the “Kickstart to Recovery” program. The demographic information suggests that all participants, with the exception of one, had previously played football at some stage in their life, many to club standard. In addition to this, a large percentage (77.8%) attend football matches as spectators and/or watch football on TV. This finding is supported by Hargreaves and Pringle (2019), who found participants held a personal meaning and previous positive experience of football in their study.

This previous connection may facilitate a reconnection with a pre-illness identity to football, which would also support qualitative findings by Mason and Holt (2012) as a benefit of participating in a football program. Meaning and value are central to recovery in mental health (Repper & Perkins, 2003). Mason and Holt (2012) found that the service users who opted to join their football group did so because they had a pre-illness identity and association with football. This unique draw to the football intervention acted as a “hook” which enticed the individual to attend initially, based on their previous positive experiences of football. It is, however, their experience during the program that encouraged them to continue to attend.

## Conclusion

This study has identified the potential benefits of the “Kickstart to Recovery” program as a resource for mental health services in Ireland. As a pilot study, with limitations highlighted below, it is recognized that the findings of this study may be attributed as factors that contributed to the changes experienced by service users and require further investigation. These findings include the indication that participants’ energy levels continued to improve throughout their participation in the program, the development of mental health management strategies for new participants supports current research on football programs for mental health services, the potential benefit of incorporating an embedded social aspect into the program, and for professionals to reflect on those referred to the program, as the participants’ previous connection to the sport may influence their motivation to partake.

Limitations of this study are related to challenges in conducting research in a “real-life” setting. Different settings made adaptions or added elements to the program that catered to the specific needs of the service users and the service. Although attempts were made to document these changes with the facilitator, it was not possible to ensure all elements were known and controlled. In addition, efforts were made to include a control group; however, given the nature of the program and practical restrictions, this was not possible. The sample size achieved also limited this study. Although the initial pre-test included 40 participants, inclusion criteria required the participant to be present for both the pre- and post-test, resulting in the sample size to fall to 27 participants.

To attempt to reduce the risk of Type I errors, the Bonferroni correction method was applied. Due to the high number of tests being performed, the *p* values decreased significantly, resulting in no statistically significant results being identified. This research included high numbers of tests, which put it at risk of increased Type II errors of identifying false negatives when applying the Bonferroni correction method (Napierala, 2012). Therefore, the results from the original *p* value were explored and discussed; however, they are recognized as a limitation of this study.

As a pilot study, this research’s strengths include the geographical area covered and use of standardized assessments. With additional local ethical approval sourced, this research covered seven sites across the Republic of Ireland. These sites were spread across the north-east, south-east, and mid-west of the country, providing a variance in environments and locations, which improves the generalization of the findings.

The questionnaire used in this research was a strength of this study as it included three standardized assessments, chosen for their good levels of validity and reliability, in addition to their ability to provide information on the aims of the study. Standardized assessments are regarded as effective in measuring subjective constructs and improve the reliability and external validity of the study (Schofield & Forrester-Knauss, 2013).

This study highlights the potential benefits the “Kickstart to Recovery” program can have for Irish mental health service users and aims to support the development of it and other similar mental health interventions.
